# *Borrelia burgdorferi* and *Anaplasma phagocytophilum* Coinfection

**DOI:** 10.3201/eid1202.050765

**Published:** 2006-02

**Authors:** Micha Loebermann, Volker Fingerle, Matthias Lademann, Carlos Fritzsche, Emil C. Reisinger

**Affiliations:** * University of Rostock Medical School, Rostock, Germany;; † Ludwig-Maximilian-Universität München, Munich, Germany

**Keywords:** ehrlichiosis, borreliosis, anaplasma, coinfection, letter

**To the Editor:** In central Europe, *Anaplasma phagocytophilum* and *Borrelia burgdorferi* are transmitted by the hard tick *Ixodes ricinus* (*1*). Acute human granulocytic ehrlichiosis (HGE) caused by *A. phagocytophilum* has rarely been documented in Europe (*2*). Typical symptoms include fever, headache, myalgia, leukopenia, thrombocytopenia, and abnormal liver function test results. The serologic prevalence ranges from 1.9% to 14% in Germany (*1*), while clinically apparent infections of HGE have not been reported.

Acute Lyme borreliosis in Europe is associated with erythema migrans (*3*), recognized in up to 90% of patients (*4*). Erythema migrans may be accompanied by systemic symptoms such as fever, fatigue, myalgia, arthralgia, headache, or stiff neck (*3**,**4*). In southern Germany, an incidence of 111 per 100,000 inhabitants has been reported (*4*).

A 60-year-old woman from northern Germany was admitted with temperature of <40°C, headache, myalgia, and generalized weakness that had begun 6 days earlier. She had noticed an erythema migrans on her right thigh 4 days before she sought treatment. At admission, a tender, 5 × 8 cm rash and a central papule were seen, but without central clearing. The clinical examination was otherwise normal. Three weeks earlier she had been on a trekking tour in Austria and Slovenia but had not been aware of any tick bites.

The leukocyte count was 3,030/μL (normal 4,000–9,000), with 65% neutrophils, 24% lymphocytes, 10% monocytes, and 1% lymphoid cells. The following results were observed: platelets 127,000/μL (normal 150,000–450,000), aspartate aminotransferase 108 U/L (normal <31), alanine aminotransferase 154 U/L (normal <34), gamma-glutamyl transferase 98 U/L (normal <38), lactate dehydrogenase 317 U/L (normal <247), alkaline phosphatase 314 U/L (normal <237), direct bilirubin 4.7 μmol/L (normal <3.4), C-reactive protein 132 mg/L (normal <5), and neopterin 30 nmol/L (normal <10). All other routine laboratory parameters were normal.

May-Grünwald-Giemsa (Fluke, Neu Ulm, Germany)–stained whole-blood smears did not show *Anaplasma* initially and during follow-up. On admission serum antibody tests were negative for *A. phagocytophilum*, *B. burgdorferi*, hepatitis A, B, and C, human herpes virus 6, herpes simplex virus 1 and 2, Epstein-Barr virus, cytomegalovirus, and tickborne encephalitis virus. Because Lyme borreliosis and possible HGE were suspected, the patient was treated with oral doxycycline 200 mg once daily for 3 weeks. Within 4 days after initiation of treatment, the patient recovered completely; thrombocytes and leukocytes had normalized. Liver enzyme levels were still elevated but had normalized at a follow-up examination 28 days later.

Four days after the initial examination, results for *Borrelia*-specific immunoglobulin M (IgM) antibodies were positive, while results for IgG antibodies remained negative (Table). Four weeks after the onset of symptoms, a test for *A. phagocytophilum*–specific IgM antibodies was positive and IgG was negative thereafter (Table). An initial EDTA blood sample that was stored frozen and examined retrospectively as well as follow-up EDTA blood samples were negative for *A. phagocytophilum* in a polymerase chain reaction (PCR) assay.

**Table:** Results of serologic tests at diagnosis and during follow-up*

Time (d) after onset of symptoms	*Anaplasma* (*Ehrlichia*) *phagocytophilum* (IFA)†		*Borrelia burgdorferi* (ELISA)‡		*Borrelia burgdorferi* (Immunoblot)§	
	IgM	IgG	IgM	IgG	IgM	IgG
6	Negative (<1:20)	Negative (<1:32)	Negative	Negative	Negative	Negative
10	ND	ND	Positive	Negative	ND	ND
28	Positive (1:40)	Negative (1:32)	Positive	Negative	Positive	Negative
107	Positive (1:20)	Negative (<1:32)	Positive	Negative	Positive	Negative
380	Negative (<1:20)	Negative (<1:32)	Equivocal	Negative	Positive	Negative

*Symptoms (fever, headache, myalgia) started 6 days before presentation. IFA, immunofluorescent assay; ELISA, enzyme-linked immunosorbent assay; Ig, immunoglobulin; ND, not done. †Genzyme Virotech, Germany. Positive titers: IgM >1:20, IgG >1:64. ‡Behring, Germany. §In-house Immunoblot, Max von Pettenkofer-Institut, Munich, Germany.

One year after initial examination, results for *Borrelia*-specific IgM antibodies were positive and results for *A. phagocytophilum*-specific antibodies were negative (Table). Although HGE has not been reported in Germany, a coinfection with *B. burgdorferi* and *A. phagocytophilum* should be considered in patients with erythema migrans and atypical changes for Lyme borreliosis such as fever, leukopenia, thrombocytopenia, and elevated liver function test results.

The patient had traveled to an area where both tickborne pathogens, *A. phagocytophilum* and *B. burgdorferi*, were endemic. Erythema migrans and antibody follow up suggested Lyme borreliosis. High fever, leukopenia, thrombocytopenia, and elevated liver enzyme levels indicated HGE. *Anaplasma* PCR was negative, possibly because blood samples were tested retrospectively after 3 months of storage at –20°C. However, a commercially available indirect fluorescent antibody assay was able to demonstrate seroconversion of HGE-specific IgM antibodies 1 month after the initial onset of symptoms. According to manufacturer's information, specificity ranged from 97.5% to 100%; sensitivity was 71.4% at 60 days after *A. phagocytophilum* infection. *A. phagocytophilum* IgG antibodies were not detected during follow-up, likely because of prompt treatment with doxycycline.

Wormser et al. (*5*) suggested that Borrelia-specific antibodies might indicate false-positive results in patients with HGE infection. Our case, however, meets criteria of a newly acquired infection with *B. burgdorferi* sensu lato, with an erythema migrans and seroconversion of *Borrelia*-specific IgM antibodies.
